# Databases and QSAR for Cancer Research

**Published:** 2007-02-15

**Authors:** Adeel Malik, Hemajit Singh, Munazah Andrabi, Syed Akhtar Husain, Shandar Ahmad

**Affiliations:** 1 Department of Biosciences, Jamia Millia Islamia University, New Delhi-110025, India

## Abstract

In this review, we take a survey of bioinformatics databases and quantitative structure-activity relationship studies reported in published literature. Databases from the most general to special cancer-related ones have been included. Most commonly used methods of structure-based analysis of molecules have been reviewed, along with some case studies where they have been used in cancer research. This article is expected to be of use for general bioinformatics researchers interested in cancer and will also provide an update to those who have been actively pursuing this field of research.

## Introduction

Bioinformatics has played a crucial role in structure based drug and target discovery, diagnosis and analysis of various diseases and their diversity. In particular there is enormous potential of its application in cancer research, which has only been partially exploited so far. Essentially all bioinformatics starts with a database and proceeds to some kind of knowledge discovery and prediction. In this article, we review bioinformatics databases and different types of quantitative structure-activity relationship (QSAR) studies, which have either been used in cancer research or have the potential of such application.

## Bioinformatics databases

Biological experiments result in useful information. This information has remained scattered in published literature, technical lab reports and patent files until not very long ago. However, there has been a tremendous effort during last couple of decades to compile, share, standardize and model biological information (e.g. Wu et al. 2003; Bairoch and Boeckmann 1991; Benson et al. 2005; Hamosh et al. 2005; Bateman et al. 2004; Boguski et al. 1993; Bauer et al. 2005; Smigielski et al. 2000; Wu et al. 2001; Berman 2000; Hulo et al. 2006; Attwood et al. 2000; Gromiha et al. 1999; Mulder et al. 2002; Pongor et al. 1992; Kanehisa and Goto 2000; Dowell et al. 2001;). There has also been relatively recent interest in improving the quality of databases, developing web-interfaces and integration of databases (Achard et al. 2001; He et al. 2005; Hanisch et al. 2002; Westbrook et al. 2002; Arauzo-Bravo and Ahmad 2005). These efforts have made it possible to know the state of the art in a given area of biology and provide a basis for what is sometimes called *in-silico* biology, as opposed to *in-vivo* and *in-vitro* biology. Some of the most widely used databases have been listed in Table 1.

The cancer research community has not remained indifferent to the importance of databases. From the big organizations such as National Cancer Institute (NCI; http://www.cancer.gov) to smaller research groups, scientists have developed databases relating to the genetics, molecular biology, microarray clinical reports and several other aspects of cancer. Table 2 lists some of the most prominent databases, which have emerged in respect of cancer research. Some of these databases are discussed below:

### Cancer Chromosomes database

(http://www.ncbi.nlm.nih.gov/entrez/query.fcgi?db=cancerchromosomes) Cancer Chromosomes integrates data from three sources: the NCI/NCBI SKY/M-FISH & CGH Database, the NCI Mitelman Database of Chromosome Aberrations in Cancer, and the NCI Recurrent Aberrations in Cancer (Knutsen et al. 2005). This is a publicly available database and can be searched for cytogenetic, clinical, and/or reference information. Similarity reports demonstrating cytogenetic and clinical relatedness at varying levels of specificity are also returned on querying this database.

### CGED (Cancer Gene Expression Database)

(http://cged.hgc.jp/cgi-bin/input.cgi) CGED is a database containing expression profiles and accompanying clinical information of breast, colorectal, and hepatocellular cancer related genes (Kato et al. 2005). The data in CGED have been obtained through collaborative efforts made at the Nara Institute of Science and Technology and Osaka University School of Medicine to identify genes of clinical importance. The expression data have been obtained by a high-throughput RT-PCR technique (adaptor-tagged competitive PCR). The data can be retrieved either using gene identifiers or by functional categories defined by Gene Ontology terms or the SwissProt annotation. Gene expression data are displayed in mosaic plots. This database also provides for the expression patterns of multiple genes, selected by names or similarity search of the patterns. The sorting function enables users for easy recognition of relationships between gene expression and clinical parameters.

### The Atlas of Genetics and Cytogenetics in Oncology and Haematology

(http://www.infobiogen.fr/services/chromcancer) The Atlas of Genetics and Cytogenetics in Oncology and Haematology is a database containing information about genes related to cancer (Huret et al. 2000). This database contains information in the form of cards on cancer related genes, chromosomal abnormalities, cancers, and cancer-prone diseases. These cards are well-structured papers, which represent the body of the Atlas. Cards on genes include data on DNA/RNA, protein, mutations, and diseases. Cards on leukemias and solid tumours include data on: clinics, cytogenetics, genes, hybrid gene and fusion protein. Cards on cancer-prone diseases include data on: inheritance mode, clinics, neoplastic risk, cytogenetics, genes and proteins, mutations. These Cards are linked to NCBI published literature database PubMed, and to other major databases (nomenclature, cartography, gene structure, transcripts, proteins, domain families, diseases, mutations, probes). This database has another component called Deep Insights and Case Reports. Deep insights are review articles related to special topics and the Case Reports section is dedicated to rare cytogenetic entities of leukemia including the associated prognosis. This database also referred to as The Atlas is part of the genome project and participates in the research in cancer epidemiology.

### Database of germline p53 mutations

(http://www.lf2.cuni.cz/projects/germline_mut_p53.htm) Somatic mutations in the p53 tumor suppressor gene are found in many human cancers (Le Roux et al. 2005). In addition, germline p53 mutations have been identified in individuals from cancer-prone families and in isolated cancer patients affected at a young age or suffering from multiple tumours (Harris 1996; Hollstein et al. 1991). A large fraction of the cancer-prone families with germline p53 mutation follow the criteria of Li-Fraumeni syndrome (LFS) (Li et al. 1988;). This syndrome is a rare familial autosomal dominant cancer syndrome characterised by early-onset sarcomas, brain tumours, premenopausal breast cancer, leukaemias and adrenocortical tumours. It is with this view that a database dedicated to p53 mutations has been developed. Genotype-phenotype correlations, compiled in this data may improve the counseling and preventive approaches in the affected families. This is a comprehensive database of those cases of germline p53 mutations for which sufficient detail is given in the literature. In addition to listing all mutations, the database includes detailed information about the families, affected individuals and their tumours. It therefore provides a powerful means for drawing correlations between various aspects of germline p53 mutations. Each p53 mutation (type of the mutation, exon and codon affected by the mutation, nucleotide and amino acid change), have been explained. In addition, it has the information on the family history of cancer, diagnosis of LFS, each affected individual (sex, generation, p53 status, from which parent the mutation was inherited) and each tumour (type, age of onset, p53 status (loss of heterozygosity and immunostaining). Each entry contains the original research article as reference(s).

### COSMIC database

(http://www.sanger.ac.uk/genetics/CGP/cosmic/) COSMIC is a database designed to store and display somatic mutation information-relevant for cancer (Forbes et al. 2006). In particular, it contains information relating to human cancers. COSMIC contains information on publications, samples and mutations implicated in cancer. It also includes samples, which have been found to be negative for mutations during screening. This allows the calculation of frequency data normalized by control frequencies for mutations in different genes in different cancer types. Samples entered include benign neoplasms and other benign proliferations, in situ and invasive tumors, recurrences, metastases and cancer cell lines. Histology and tissue ontology has also been created in this database. All mutations are mapped to a single version of each gene. The data can be queried by tissue, histology or gene and displayed as a graph, as a table or exported to other formats.

### EHCO database

(http://ehco.nchc.org.tw) EHCO (Encyclopedia of Hepatocellular Carcinoma genes Online) is an integrative database for HCC (hepatocellular carcinoma) research. It carries gene annotations collected by computer-assisted mining, manual curation, and extraction from public databases. Currently EHCO contains information for about 3500 HCC-related genes. Various entries in this database can be compared online. Detailed annotations for particular genes, including sequence, ontology, cited literature, and expression profiles are also available.

### Human p53 database

(http://metalab.unc.edu/dnam/mainpage.html) A collection of databases relating to p53 gene mutations, lacI and lacZ is available on this website (Cariello et al. 1994; Cariello et al. 1996). There are nearly 6000 entries corresponding to p53, 200 for lacZ and 1500 of lacI. In addition 1500 transgenic and 8000 bacterial entries are also included. A software for analysis of the databases is also included. Each database has a separate software analysis program. All these databases include information about mutations such as base position, the nature of the mutation, amino acid position, molecular weight and the name of mutant amino acid, the local sequence around a mutation and literature citation as the source of listed information. Information specific to the p53 database includes cancer type, cell origin, loss of heterozygosity.

### IARC TP53 Database

(http://www.p53.iarc.fr/index.html) The IARC TP53 Database compiles data on human somatic and germline TP53 genetic variations that are reported in the published literature. (Olivier et al. 2002; Hernandez-Boussard 1999; Hainaut et al. 1997; Hainaut et al. 1998 ; Hollstein et al. 1994, Hollstein et al. 1996). With over 18,500 somatic and 225 germline mutations and 1,000 citations in the world literature, this database is now recognized as a major source of information on TP53 mutation patterns in human cancer. It can be searched and analyzed online and is useful to draw hypotheses on the nature of the molecular events involved in TP53 mutagenesis and on the natural history of cancer.

### ITTACA Gene expression and clinical database

(http://bioinfo-out.curie.fr/ittaca/) ITTACA is a database of microarray experimental results and clinical information retrieved form published papers (Elfilali et al. 2006). It contains information on breast carcinoma, bladder carcinoma, and uveal melanoma. Online service also allows some basic statistical analysis of the database such as the comparison of expression distribution profiles, tests for differential expression, and patient survival analyses.

### The Mouse Tumor Biology Database (MTB)

(http://www.informatics.jax.org) MTB database compiles and shares information about tumor frequency, genetics, and pathology in genetically denned mice (i.e., transgenics, targeted mutations, and inbred strains) (Bult et al. 2001). The database collects crucial information about incidence of different types of tumors in different strains, mutations relating to specific genes and tumors corresponding to them, which have been reported in medical journals. Existing standards for anatomy, tumor names, gene names, and strain names are well enforced, enabling direct links to information across MTB entries and to other relevant databases.

### The Tumor Gene Family Databases (TGDBs)

(http://condor.bcm.tmc.edu/ermb/tgdb/tgdf.html) TGDB is made up of two databases viz. Oral Cancer Gene Database (OrCGDB) and Breast Cancer Gene Database (BCGD). Both these databases contain information on a mechanism of oncogenic activation, regulation, frequency of involvement in various tumor types, and chromosomal location for the genes involved in cancer (e.g. proto-oncogenes and tumor supressor genes). Data about the encoded proteins includes the cell type in which they are found, subcellular location, DNA, protein, and ligand binding, role in development, and normal biochemical function.

### QSAR and in-silico analysis of molecular recognition

Once the molecular mechanism and the chemistry of a disease is understood, the next crucial task is to find a suitable cure for it. Atypical requirement is to find a suitable drug target and the drug itself (Brooijmans and Kuntz 2003). Target discovery draws much on bioinformatics tools today and in case of cancer the DNA and protein molecules both can be potential targets for drugs (Choudhary et al. 2005; Bandyopadhyaya et al. 2005; Bhongade et al. 2004; Asseffa et al. 2003; Gellert et al. 2005; Khaleque et al. 2006; Yao et al. 2005; McColl et al. 2005).

Drug discovery is a complex, expensive and very time-consuming exercise, as there is no single systematic way to automatically discover a drug even when the disease and targets have been well understood (Dixit and Mitra 2002).

**Figure 1.**
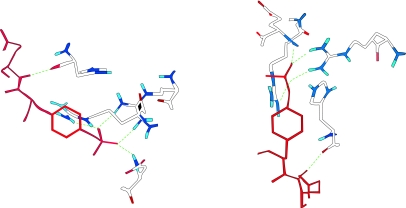
Identical A & B Chain Residues of 1A1E in complex with its ligand (ACE-PTR-GLU-DIY). Ligand in red.

There may be millions of candidate molecules if in-silico filtering is not performed. Experiments cannot be performed on such large number of drug candidates due to prohibitive costs both in terms of time and money. Quantitative structure-activity relationship (QSAR) studies form the center stage when a protein (typically an enzyme) is the target and there is a need to find a suitable molecule, which can control (inhibit) the activity of its target. The basic principle of such a study is the structure-dependence of chemical activity. QSAR has existed much longer than the first popularity of computers, because chemical structure has always been able to explain at least some aspects of chemical properties. However, with the availability of powerful computers and high quality databases of molecular libraries and interactions have made QSAR an essential component of drug discovery today. Role of structure in determining the activity of a chemical compound is illustrated in an example of protein-ligand complex in Fig 1.

QSAR based (in-silico) analysis may be better regarded as an exercise to screen or filter drug candidates, before they are subjected to more intensive calculations such as docking or an experimental measurement of activity (in-vitro) and finally under real conditions (in-vivo). Many times this step will pick up a dozen of drug candidate from a library of millions of well-studied molecules. Traditional QSAR is specific to a particular target or enzyme and all the screening is performed on drug candidates (ligand molecules). These ligand molecules are very diverse and in order to screen them suitably, we need to describe their structure as well as chemical nature. This leads to the issue of finding descriptors of molecular properties of ligands and drugs. Hundreds of molecular properties or descriptors are used to represent molecules (Labute 2000; Xue and Bajorath 2000; Wildman and Crippen 2002; Gozalbes et al. 2002).

These properties may be purely geometric, topological, electromagnetic, classical and quantum-mechanical. Often, predicting activity of a protein-ligand combination if the descriptors of the ligand are known carries out this screening. Regression techniques such as Principal Component Analysis (PCA), Neural Network and Multi-variate correlation are the major techniques used for this purpose. In the following we review some of these techniques and special reference will be wherever a successful application to cancer has been reported.

A large number of molecular descriptors are available and used (Todeschini and Consonni; Labute 2000; Wildman and Crippen 2002; Hansch et al. 1995; Basak et al. 1980; Gozalbes et al. 2002; Pirard and Picket 2000; Basak et al. 1981; Basak et al. 1982; Kier and Hall 1999; Raevsky 1999; Xue and Bajorath 2000). Molecular descriptors used in QSAR for a unique representation and identification of ligand molecules, which are likely to be drug candidates, may be classified as follows:

**Constitutional descriptors such as** molecular weight, van der Waals volume, electronegativities, polarizability, number of atoms, non-H atoms, number of H bonds, multiple bonds, bond orders, aromatic ratio, number of rings, number of double and triple bonds, aromatic bonds, 3 different types of (n-membered) rings, benzene-like rings.

**Topological descriptors such as** total structure connectivity index, Pogliani index, ramification index, polarity number, average vertex distance degree, mean square distance index (Balaban), Schultz Molecular Topological Index (MTI), square reciprocal distance sum index, quasi-Wiener index (Kirchhoff number), spanning tree number, hyper-distance-path index, reciprocal hyper-distance-path index, detour index, hyper-detour index, reciprocal hyper-detour index, distance/detour index, all-path Wiener index, Wiener-type index from Z weighted distance matrix (Barysz matrix), molecular electrotopological variation, E-state topological parameter, Kier symmetry index eccentricity, mean distance degree deviation, unipolarity, centralization, variation.

**Walk and path counts** such as molecular walk counts, total walk count, self-returning walk counts, molecular path counts, molecular multiple path counts, total path count, conventional bond-order ID number, Randic ID number, Balaban ID number, ratio of multiple path count over path count, difference between multiple path count and path count.

**Connectivity indices such as** connectivity indices, average connectivity indices, valence connectivity indices, average valence connectivity indices, solvation connectivity indices, modified, reciprocal distance Randic-type index, reciprocal distance squared Randic-type index.

**Information indices such as** information index on molecular size, total information index of atomic composition, mean information index on atomic composition, mean information content on the distance equality, mean information content on the distance magnitude, mean information content on the distance degree equality, mean information content on the distance degree magnitude, total information content on the distance equality, total information content on the distance magnitude, mean information content on the vertex degree equality, mean information content on the vertex degree magnitude, graph vertex complexity index, graph distance complexity index (log), Balaban U index, Balaban V index, Balaban X index, Balaban Y index Basak indices of neighborhood symmetry.

**2D autocorrelations** Broto-Moreau autocorrelations of a topological structure, Moran autocorrelations, Geary autocorrelations.

**Edge adjacency indices** edge connectivity index of order 0, edge connectivity index of order 1 eigenvalues from edge adj. matrix weighted by edge degrees, eigenvalues from edge adj. matrix weighted by dipole moments, eigenvalues from edge adj. matrix weighted by resonance integrals spectral moments from edge adj. matrix, spectral moments from edge adj. matrix weighted by edge degrees, spectral moments from edge adj. matrix weighted by dipole moments, spectral moments from edge adj. matrix weighted by resonance integrals.

**Eigenvalue-based indices** Lovasz-Pelikan index (leading eigenvalue), leading eigenvalue from Z weighted distance matrix (Barysz matrix), leading eigenvalue from mass weighted distance matrix, leading eigenvalue from van der Waals weighted distance matrix, leading eigenvalue from electro-negativity weighted distance matrix, leading eigenvalue from polarizability weighted distance matrix.

**Geometrical descriptors** 3D-Wiener index, 3D-Balaban index, 3D-Harary index average geometric distance degree, D/D index, average distance/distance degree gravitational index G1, gravitational index G2 (bond-restricted), radius of gyration (mass weighted), span R, average span R.

**Functional group counts** terminal primary C(sp3), total secondary C(sp3), total tertiary C(sp3), total quaternary C(sp3), ring secondary C(sp3), ring tertiary C(sp3), ring quaternary C(sp3) aromatic C(sp2), unsubstituted benzene C(sp2), substituted benzene C(sp2), non-aromatic conjugated C(sp2), terminal primary C(sp2), aliphatic secondary C(sp2), aliphatic tertiary C(sp2), allenes groups, terminal C(sp), non-terminal C(sp) cyanates (aliphatic), cyanates (aromatic), isocyanates (aliphatic), isocyanates (aromatic), thiocyanates (aliphatic), thiocyanates (aromatic), isothiocyanates (aliphatic), isothiocyanates (aromatic).

**Charge descriptors** maximum positive charge, maximum negative charge, total positive charge, total negative charge, total absolute charge (electronic charge index – ECI), mean absolute charge (charge polarization), total squared charge, relative positive charge, relative negative charge, submolecular polarity parameter, topological electronic descriptor, topological electronic descriptor (bond resctricted), partial charge weighted topological electronic descriptor, local dipole index.

**Molecular properties** unsaturation index hydrophilic factor Ghose-Crippen molar refractivity topological polar and non-polar surface area.

Many more descriptors may be calculated and comprehensive lists can be found. A comprehensive review of molecular descriptors is presented by Karelson (2000). Many free and commercial software also provide a current list of descriptors (e.g. http://www.talete.mi.it/products/dragon_molecular_descriptors.htm and http://preadmet.bm-drc.org/preadmet/query/query1.php, from where, list of many of the above descriptors is compiled.). An excellent coverage of issues and topics related to QSAR is also provided in a text book by Gasteiger and Engel (2003).

After the descriptors of molecules have been calculated, redundant descriptors are removed using Principal Component Analysis or Multivariate analysis (Jolliffe 1986: Xue and Bajorath 2000). Many commercial and some free software programs are now available which may be used to calculate some of the descriptors and/or develop a QSAR model using them. Some of these programs are listed in Table 3. These softwares can give few key descriptors (such as 5 descriptors in Molinspiration) or a very large number of them (e.g. DRAGON gives more than 1500 descriptors), which will need to be reduced by some analysis.

Cancer researchers have frequently used these methods for a systematic filtering of potential drug candidates or for generalizing principles governing the choice of ligands that prefer to bind to a particular family of proteins in a selective and competitive way. Several aspects of cancer have been studied using QSAR techniques. Classical efforts at using QSAR for cancer drug research date back to 1970s (e.g. Hansch 1979). Antitumour drugs have remained a regular subject of investigation using QSAR (Ren and Lien 2004). During that time, focus was to discover drugs for chemotherapy. As cases of multidrug resistance were observed, a need to have alternative medicine for the same action were felt. Thus, a large number of researchers have focused on multidrug resistance in regards to chemotherapy and employed QSAR as a means to solve this problem. For example Breier et al. (2000) have studied multidrug resistance (MDR) for L1210/VCR-1 and L1210/VCR-2 cell lines in regards to leukemia treatment. They related the developed adaptation and drug resistance to structure descriptors of drugs viz. binding energy, molecular weight, pKa, log P etc. Klopman et al. (1997) have studied 609 diverse compounds to understand the drug resistance in P388/ADR resistant cell lines. In this study they identified several structural characteristics of MDR such as log P and graph index. More advanced techniques of QSAR such as Comparative Molecular Simillarity Index Analysis (CoMSIA) have been used to study antiviral and anticancer drugs targeting Thymidine Kinase (e.g. Bandyopadhyaya et al. 2005, Bhongade and Gadad 2004). Principle of CoMSIA is the alignment and comparison of drug molecules by comparing their similarity indices (selected descriptors). A similar approach, called Comparative Molecular Field Analysis (CoMFA) focuses on molecular field descriptors for this purpose (Cramer et al. 1988). Epidermal Growth Factor Receptors (EGFR) are one of the most popular class of proteins studied by QSAR method. Assefa et al. (2003) have used CoMFA for such a study and concluded that electrostatics and hydrophobicity descriptors play the most important role in EPGR target binding. Similarly, electrotopological state atom (ETSA) indices have been shown to play the most important role in anti tumour effect of pyridoacridine ascididemin analagues (Debnath et al. 2003). Thus, if a drug is available for chemotherapy and more such drugs are required to have redundancy against drug resistance, previously known successful drug/inhibitor is compared with a large data set of diverse molecules and those having their molecular indices (CoMSIA), or molecular fields (CoMFA) similar to that drug are picked up for potential use. Most recent QSAR related cancer studies have focused on genomic aspects of cancer related drug discovery (Workman 2001, Jung et al. 2003). This allows for individual prescriptions based on the genetic makeup of the patient. Thus, the possibility of having a large number of drugs having similar inhibitory ability but diverse genetic response opens a myriad of possibilities for cancer related research for peoples and individuals.

## Summary

A number of databases directly and indirectly useful for cancer research have been reviewed. QSAR techniques and its application to cancer research have been outlined.

## Figures and Tables

**Table 1.** General Bioinformatics Databases.

**Major sequence repositories**		
DNA Data Bank of Japan (DDBJ)	http://www.ddbj.nig.ac.jp	All known nucleotide and protein sequences; International Nucleotide Sequence Database Collaboration
EMBL Nucleotide Sequence Database	http://www.ebi.ac.uk/embl.html	All known nucleotide and protein sequences; International Nucleotide Sequence Database Collaboration
GenBank	http://www.ncbi.nlm.nih.gov/	All known nucleotide and protein sequences; International Nucleotide Sequence Database Collaboration
NCBI Reference Sequence Project	http://www.ncbi.nlm.nih.gov/RefSeq/	Non-redundant collection of naturally-occurring biological molecules
Ensembl	http://www.ensembl.org/	Annotated information on eukaryotic genomes
UCSC Genome Browser	http://genome.ucsc.edu/	Genome assemblies and annotation
UniGene	http://www.ncbi.nlm.nih.gov/UniGene/	Non-redundant, gene-oriented clusters
**Protein Databases**		
CSDBase	http://www.chemie.uni-marburg.de/~csdbase/	Cold shock domain-containing proteins
DExH/D Family Database	http://www.helicase.net/dexhd/dbhome.htm	DEAD-box, DEAH-box and DExH-box proteins
Endogenous GPCR List	http://www.tumor-gene.org/GPCR/gpcr.html	G protein-coupled receptors; expression in cell lines
EXProt	http://www.cmbi.Kun.nl/EXProt/	Proteins with experimentally-verified function
GenProtEC	http://genprotec.mbl.edu	*E. coli* K-12 genome, gene products and homologs
Histone Database	http://research.nhgri.nih.gov/histones/	Histone and histone fold sequences and structures
HIV Molecular Immunology Database	http://hiv-web.lanl.gov/content/immunology/index	HIV epitopes
HIV RT and Protease Sequence Database	http://hivdb.stanford.edu	HIV reverse transcriptase and protease sequences
Homeodomain Resource genomic	http://genome.nhgri.nih.gov/homeodomain/	Homeodomain sequences, structures and related genetic and genomic information
HUGE	http://www.kazusa.or.jp/huge/	Large (>50 kDa) human proteins and cDNA sequences
IMGT	http://imgt.cines.fr	Immunoglobulin, T cell receptor and MHC sequences from human and other vertebrates
IMGT/HLA	http://www.ebi.ac.uk/imgt/hla/	Polymorphic sequences of human MHC and related genes
IMGT/MHC Database	http://www.ebi.ac.uk/ipd/mhc/index.html	Major histocompatibility complex sequences
InBase	http://www.neb.com/neb/inteins.html	All known inteins (protein splicing elements): properties, sequences, bibliography
InterPro	http://www.ebi.ac.uk/interpro	Protein families and domains
LGICdb	http://www.ebi.ac.uk/compneur-srv/LGICdb/LGICdb.php	Ligand-gated ion channel subunit sequences
Nuclear Protein Database (NPD)	http://npd.hgu.mrc.ac.uk	Proteins localized in the nucleus
NRMD	http://www.receptors.org/NR/	Nuclear receptor superfamily
NUREBASE	http://www.ens-lyon.fr/LBMC/laudet/nurebase.html	Nuclear hormone receptors
ooTFD	http://www.ifti.org/ootfd	Transcription factors and gene expression
PANTHER	http://www.pantherdb.org/	Gene products organized by biological function
Peptaibol	http://www.cryst.bbk.ac.uk/peptaibol/home.shtml	Peptaibol (antibiotic peptide) sequences
Phospho.ELM	http://phospho.elm.eu.org/	Protein phosphorylation sites
PKR	http://www.kinasenet.org/pkr/Welcome.do	Protein kinase sequences, enzymology, genetics and molecular and structural properties
Prolysis	http://delphi.phys.univ-tours.fr/Prolysis/	Proteases and natural or synthetic protease inhibitors
Protein Information Resource (PIR)	http://pir.georgetown.edu	Comprehensive, annotated, non-redundant protein sequence databases
ProtoNet	http://www.protonet.cs.huji.ac.il/	Hierarchical clustering of protein sequences
RTKdb	http://pbil.univ-lyon1.fr/RTKdb/	Receptor tyrosine kinase sequences
SEVENS	http://sevens.cbrc.jp	7-transmembrane helix receptors
SWISS-PROT/TrEMBL	http://www.expasy.org/sprot	Curated protein sequences
TIGRFAMs	http://www.tigr.org/TIGRFAMs	Functional identification of proteins
trEST, trGEN, Hits	http://hits.isb-sib.ch	Hypothetical protein sequences
**Structure**		
ASTRAL	http://astral.stanford.edu/	Sequences of domains of known structure, selected subsets and sequence-structure correspondences
BioMagResBank acids	http://www.bmrb.wisc.edu/	NMR spectroscopic data from proteins, peptides, and nucleic acids
CATH	http://www.biochem.ucl.ac.uk/bsm/cath_new	Protein domain structures
CKAAPs DB	http://ckaaps.sdsc.edu/perl/browser.pl	Structurally-similar proteins with dissimilar sequences
CSD	http://www.ccdc.cam.ac.uk/products/csd/	Crystal structure information for organic and metal organic compounds
Database of Macromolecular Movements	http://bioinfo.mbb.yale.edu/Mol-MovDB/	Descriptions of protein and macromolecular motions, including movies
Decoys ‘R’ Us	http://dd.stanford.edu/	Computer-generated protein conformations based on sequence data
DSMM	http://projects.eml.org/mcm/database/dsmm	Database of Simulated Molecular Motions
Gene3D	http://cathwww.biochem.ucl.ac.uk:8080/Gene3D/	Precalculated structural assignments for genes within whole genomes
GTOP	http://spock.genes.nig.ac.jp/~genome/gtop.html	Protein fold predictions from genome sequences
HIC-Up	http://alpha2.bmc.uu.se/hicup/	Structures of small molecules (‘hetero-compounds’)
HSSP	http://www.sander.ebi.ac.uk/hssp/	Structural families and alignments; structurally-conserved regions and domain architecture
LPFC	http://smi-web.stanford.edu/projects/helix/LPFC/	Library of protein family core structures
MMDB linked	http://www.ncbi.nlm.nih.gov/Structure/	All experimentally-determined three-dimensional structures, linked to NCBI Entrez
ModBase	http://modbase.compbio.ucsf.edu/modbase-cgi-new/index.cgi	Annotated comparative protein structure models
NDB	http://ndbserver.rutgers.edu/	Nucleic acid-containing structures
NTDB	http://ntdb.chem.cuhk.edu.hk^¶^	Thermodynamic data for nucleic acids
PALI	http://pauling.mbu.iisc.ernet.in/~pali	Phylogeny and alignment of homologous protein structures
PASS2	http://caps.ncbs.res.in/campass/pass.html	Structural motifs of protein superfamilies
PDB	http://www.pdb.org/	Structure data determined by X-ray crystallography and NMR
PDB-REPRDB	http://mbs.cbrc.jp/pdbreprdb-cgi/reprdb_menu.pl	Representative protein chains, based on PDB entries
PDBsum	http://www.ebi.ac.uk/thornton-srv/databases/pdbsum/	Summaries and analyses of PDB structures
ProTherm	http://gibk26.bse.kyutech.ac.jp/jouhou/therm/protherm.html	Thermodynamic data for Pro-wild-type and mutant proteins
PSSH	http://srs3d.ebi.ac.uk/	Alignments between protein sequences and tertiary structures
RNABase	http://www.rnabase.org	RNA-containing structures from PDB and NDB
SCOP	http://scop.mrc-lmb.cam.ac.uk/scop	Familial and structural protein relationships
SCOR	http://scor.lbl.gov	RNA structural relationships
Sloop	http://www-cryst.bioc.cam.ac.uk/~sloop/	Classification of protein loops
Structure-Superposition Database	http://ssd.rbvi.ucsf.edu	Pairwise superposition of TIM-barrel structures
SUPERFAMILY	http://supfam.org	Assignments of proteins to structural superfamilies
**Retrieval Systems and Database Structure**		
TESS	http://www.cbil.upenn.edu/cgi-bin/tess/tess	Transcription element search system
Virgil	http://www.infobiogen.fr/services/virgil^¶^	Database interconnectivity

**Table 2.** Cancer related bioinformatics databases.

Database Name	URL	Description
Atlas of Genetics and Cytogenetics in Oncology and Haematology	http://www.infobiogen.fr/services/chromcancer/	Cancer-related genes, chromosomal abnormalities in oncology and haematology, and cancer-prone diseases
Cancer Chromosomes	http://www.ncbi.nlm.nih.gov/entrez/query.fcgi?db=cancerchromosomes	Cytogenetic, clinical and reference information on cancer-related aberrations
CGED	http://cged.hgc.jp/cgi-bin/input.cgi	Cancer gene expression database
COSMIC	http://www.sanger.ac.uk/genetics/CGP/cosmic/	Catalogue of somatic mutations in cancer: sequence data, samples and publications
Germline p53 Mutations	http://www.lf2.cuni.cz/win/projects/p53.htm	Mutations in germline_mut_ human tumor and cell line p53 gene
IARC TP53 Database	http://www.p53.iarc.fr/index.html	Human TP53 somatic and germline mutations
MTB	http://tumor.informatics.jax.org/mtbwi/index.do	Mouse tumor biology database: tumor types, genes, classification, incidence, pathology
OncoMine	http://www.oncomine.org/	Cancer microarray data by gene or cancer type
Oral Cancer Gene Database	http://www.tumor-gene.org/Oral/oral.html	Cellular and molecular data for genes involved in oral cancer
RB1 Gene Mutation DB	http://www.verandi.de/joomla/	Mutations in the human retinoblastoma (RB1) gene
RTCGD	http://rtcgd.ncifcrf.gov/	Mouse retroviral tagged cancer gene database
SNP500Cancer	http://snp500cancer.nci.nih.gov	Re-sequenced SNPs from 102 reference samples
SV40 Large T-Antigen Mutants	http://www.pitt.edu/pipaslab/^¶^	Mutations in SV40 large tumor antigen gene
Tumor Gene Family Databases	http://www.tumor-gene.org/tgdf.html	Cellular, molecular and biological data about genes involved in various cancers

¶ These sites could not be opened at the time of revising the manuscript.

**Table 3.** Available QSAR and molecular descriptor programs.

Name of the software	Brief Description	URL/Reference
PEST	Shape properties, Wavelet decomposition properties, Electrostatic potential, electronic kinetic energy density etc.	*C. Matthew Sundling, N. Sukumar* and *Curt Breneman* Rensselaer Polytechnic Institute http://www.chem.rpi.edu/chemweb/recondocpest.html
Pharma Algorithm’s QSAR Builder	QSAR and QSPR modeling; Excess molar refraction, H-bond acidity, H-donor capability, H-bond basicity, H-acceptor capability Hexadecane/gas partition coefficient, LogP partition coefficient, TPSA - topological polar surface area	**Hugo Kubinyi,** Professor of Pharmaceutical Chemistry at the University of Heidelberg, Germany http://apalgorithms.com/qsar_builder.htm
Bioreason	QSAR, QPSR	**Commercial by Bioreason**
ClassPharmer		http://www.bioreason.com
ChemTK	Molecule design, descriptors and modeling	http://www.sageinformatics.com/chemtk.html
Molinspiration toolkit	Java Based software and free online calculations of fragments and basic properties/descriptors.	http://www.molinspiration.com
ShapeSig	Shape desciptors, and statistical analysis	http://histidine.umdnj.edu/~shape/index.php
Cerius (QSAR module)	Modeling and QSAR, includes MOPAC Quantum mechanical calculations, alignments etc.	http://www.accelrys.com
CODESSA	QSAR program	http://www.semichem.com/codessa/default.php
HASL	3D QSAR	http://www.bio.com/store/product.jhtml?id=prod300024
QTRFIT	Rigid body superposition	http://www.osc.edu/PET/CCM/skeleton/software/tested/source/qtrfit/qtrfit.html
DRAGON	1664 molecular descriptors	http://www.talete.mi.it/main_net.htm
